# Bradykinin-Induced Sensitization of Transient Receptor Potential Channel Melastatin 3 Calcium Responses in Mouse Nociceptive Neurons

**DOI:** 10.3389/fncel.2022.843225

**Published:** 2022-04-13

**Authors:** Marc Behrendt, Hans Jürgen Solinski, Martin Schmelz, Richard Carr

**Affiliations:** Department of Experimental Pain Research, MCTN, Medical Faculty Mannheim, University of Heidelberg, Mannheim, Germany

**Keywords:** mouse dorsal root ganglion (DRG) neurons, inflammatory mediators and pain, diacylglycerol (DAG) kinase/lipase, G protein-coupled receptor (GPCR) intracellular signaling, transient receptor potential (TRP) channel melastatin 3 (TRPM3)

## Abstract

TRPM3 is a calcium-permeable cation channel expressed in a range of sensory neurons that can be activated by heat and the endogenous steroid pregnenolone sulfate (PS). During inflammation, the expression and function of TRPM3 are both augmented in somatosensory nociceptors. However, in isolated dorsal root ganglion (DRG) neurons application of inflammatory mediators like prostaglandins and bradykinin (BK) inhibit TRPM3. Therefore, the aim of this study was to examine the effect of preceding activation of cultured 1 day old mouse DRG neurons by the inflammatory mediator BK on TRPM3-mediated calcium responses. Calcium signals were recorded using the intensity-based dye Fluo-8. We found that TRPM3-mediated calcium responses to PS were enhanced by preceding application of BK in cells that responded to BK with a calcium signal, indicating BK receptor (BKR) expression. The majority of cells that co-expressed TRPM3 and BKRs also expressed TRPV1, however, only a small fraction co-expressed TRPA1, identified by calcium responses to capsaicin and supercinnamaldehyde, respectively. Signaling and trafficking pathways responsible for sensitization of TRPM3 following BK were characterized using inhibitors of second messenger signaling cascades and exocytosis. Pharmacological blockade of protein kinase C, calcium–calmodulin-dependent protein kinase II and diacylglycerol (DAG) lipase did not affect BK-induced sensitization, but inhibition of DAG kinase did. In addition, release of calcium from intracellular stores using thapsigargin also resulted in TRPM3 sensitization. Finally, BK did not sensitize TRPM3 in the presence of exocytosis inhibitors. Collectively, we show that preceding activation of DRG neurons by BK sensitized TRPM3-mediated calcium responses to PS. Our results indicate that BKR-mediated activation of intracellular signaling pathways comprising DAG kinase, calcium and exocytosis may contribute to TRPM3 sensitization during inflammation.

## Introduction

The physiological role of somatosensory nociceptors is to detect potentially and overtly harmful stimuli in order to evoke immediate protective reflexes and pain sensations. Tissue damage and inflammation increase tissue vulnerability and can lead to a decrease in the activation threshold. Reductions in nociceptor activation threshold may arise through a range of sensitization processes including post-translational modifications of ion channels and altered protein expression. These processes may involve neuroactive agents released from various cell types at the site of inflammation (Willemen and Eijkelkamp, 2020). Among the pain-related ion channels expressed in nociceptors and sensitized under inflammatory conditions are several members of the family of transient receptor potential (TRP) channels, in particular TRPV1, TRPA1, and TRPM3 (Silverman et al., 2020).

Members of the TRP family form tetrameric non-selective, mostly calcium-permeable, cation channels. In mammals, many TRPs serve as molecular sensors and signal transduction conduits for a variety of chemical and physical stimuli (Flockerzi and Nilius, 2014). TRP channels can also be regulated synergistically by intracellular signaling cascades. For example, both the expression and sensitivity of the heat and capsaicin receptor TRPV1 (Caterina et al., 1997) can be increased through intracellular signaling pathways involving phospholipase C (PLC) and initiated by inflammatory mediators such as bradykinin (BK), prostaglandin E2, and nerve growth factor (NGF) (Huang et al., 2006; Mathivanan et al., 2016). Similarly, TRPA1, the receptor for allyl isothiocyanate, the pungent component of mustard oil, can be sensitized by signaling cascades secondary to activation of BK receptors (BKRs) (Katanosaka et al., 2008; Wang S. et al., 2008; Forster et al., 2009), prostaglandin receptors (Dall’Acqua et al., 2014) and glutamate receptors (Masuoka et al., 2016). TRP channels are thus direct sensors of physicochemical stimuli and their sensitivity can be indirectly regulated by other surface receptors on their host cell.

TRPM3 is expressed in dorsal root ganglion (DRG) neurons and, together with TRPV1 and TRPA1, is implicated in the transduction of heat stimuli (Vriens et al., 2011; Vandewauw et al., 2013, 2018). TRPM3 proteins form cation channels activated by both heat and the endogenous steroid pregnenolone sulfate (PS) (Grimm et al., 2003; Wagner et al., 2008; Drews et al., 2014; Demirkhanyan et al., 2016). TRPM3 is also involved in the development of inflammatory heat hypersensitivity (Vriens et al., 2011) and TRPM3 expression is increased and channel function is augmented following inflammation when the levels of inflammatory mediators like prostaglandins and BK are high (Mulier et al., 2020). In TRPM3-deficient mice, the development of inflammatory heat hyperalgesia is impaired and pharmacological inhibition of TRPM3 is sufficient to eliminate the development of inflammatory heat hyperalgesia in wild type mice (Vriens et al., 2011; Straub et al., 2013; Alkhatib et al., 2019). Furthermore, TRPM3 is upregulated during bladder inflammation in afferent bladder neurons (Vanneste et al., 2021). Moreover, the number of nociceptors co-expressing TRPM3 and the two other heat-activated TRP channels TRPA1 and TRPV1 increased in a mouse model of complete Freund’s adjuvant-induced peripheral inflammation, and, interestingly, TRPM3 inhibition reduced TRPA1- and TRPV1-mediated calcium responses in inflamed tissue (Mulier et al., 2020).

In isolated DRG neurons, TRPM3 calcium responses to PS can be inhibited reversibly by co-application of agonists for Gi-coupled G protein-coupled receptors (GPCRs) including μ-opioid, somatostatin and GABA-B receptors (Badheka et al., 2017; Dembla et al., 2017; Quallo et al., 2017). Similarly, co-application of agonists for Gs-coupled prostaglandin receptors and Gq-coupled BKRs both suppress calcium signals to PS (Alkhatib et al., 2019). However, if application of carbachol, a Gq-coupled agonist, precedes TRPM3 activation in HEK-293 cells, TRPM3-dependent calcium influx can be enhanced (Lee et al., 2003). This suggests that it is the activation state of TRPM3 at the time of GPCR stimulation that determines whether TRPM3 will be inhibited or sensitized by G protein signaling cascades. To explore this, agonists of TRPM3 and Gq-coupled BKRs were applied to DRG neurons over discrete, non-overlapping time periods. To examine the time course of this effect, cultured 1 day old DRG neurons were exposed to the inflammatory mediator BK before applying PS. Under these conditions, TRPM3-mediated calcium responses to PS were enhanced by preceding application of BK. This sensitization of TRPM3 pursuant to BKR activation is attributed to pathways involving calcium store release, diacylglycerol kinase (DAGK) and exocytosis.

## Results

**FIGURE 1:**
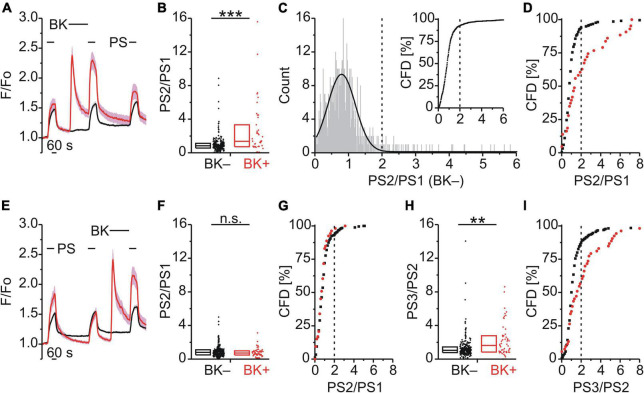
Bradykinin sensitizes calcium responses to pregnenolone sulfate in DRG neurons. **(A–D)** Calcium signals over time from mouse DRG neurons **(A)** during three applications of pregnenolone sulfate (PS; 40 μM) with bradykinin (BK; 0.5 μM) applied between PS1 and PS2. Cells that showed an increase in calcium to BK are indicated in red (BK+; **A,B,D**, 43 neurons) and those that did not in black (BK–; **A,B,D**, 216 neurons). To assess the effect of BK on PS-evoked calcium response amplitude the ratio PS2–PS1 amplitude (PS2/PS1) was determined for all cells that did not respond to BK (**C**, 587 neurons, from Figures 1, 2D–F). This distribution of PS2/PS1 ratios was fit by a Gaussian function (**C**, solid black line) and the fit parameters were used to define a threshold corresponding to the mean plus three standard deviations (**C**, broken vertical line at 2.0). Cells with ratio values above threshold were considered to be sensitized and plotting PS2/PS1 values cumulatively (**C**, inset; CFD, cumulative frequency distribution) illustrates that in this control group 7.5% of values were above this threshold. For application of BK between PS1 and PS2, PS2/PS1 ratios are shown as a simple distribution (**B**, boxes indicate quartiles and centerline median) and as CFD **(D)**. Each dot-like symbol in **(B)**, (**C**, inset), and **(D)** represents the ratio value calculated for a single, individual cell. **(E–I)** Calcium signals over time **(E)** from mouse DRG neurons during repeat application of PS with BK applied between PS2 and PS3 (BK+, 53 neurons; BK–, 265 neurons). Control PS2/PS1 ratios are shown as a simple distribution **(F)** and CFD **(G)**. PS3/PS2 ratios are shown as a simple distribution **(H)** and CFD **(I)**. Two ratio values (“35.4” and “28.7”) of the BK– group were not shown in **(H)** for better visibility, but were included in the analysis. Values derive from sixteen measurements with cells from two mice. BK+ and BK– groups **(B,F,H)** were compared with Mann-Whitney *U*-tests. **P < 0.01; ***P < 0.001; n.s., not significant.

We used calcium imaging as a functional assay for TRPM3 in mouse DRG neurons. In order to examine the effect of precedent GPCR activation on calcium responses to the TRPM3 agonist PS we used a simple protocol comprising three successive PS applications. In Figure 1A, calcium signals to repeat PS application are shown over time as mean calcium intensity with standard errors indicated by the shading (Figure 1A). The effect of intervening GPCR activation was quantified by determining the calcium response ratio (PS2/PS1) between the second PS application (PS2), i.e., after the GPCR agonist, and the first PS application (PS1), before the GPCR agonist.

### Bradykinin Produced a Calcium Response and Increased the Calcium Response Amplitude to Subsequent Pregnenolone Sulfate

BK, a Gq-coupled agonist, was used to examine modulation of TRPM3 by an inflammatory mediator (Figure 1A). BK evoked a calcium response in 16.6% of DRG neurons that also responded with a calcium increase to PS (43/259; BK+; red trace; Figure 1A). Interestingly, within this subset of BK+ neurons, the response to the second application of PS (PS2) was larger than the PS2 response in those cells that did not respond to BK (BK–; black trace; Figure 1A). This effect was quantified by determining the ratio of calcium response amplitudes between the second (PS2) and the first (PS1) PS application (Figure 1B). Comparing these two groups indicated that the PS2/PS1 ratio for BK+ neurons was significantly higher than for BK– neurons (*p* < 0.001, Mann-Whitney U; Figure 1B). From the distribution of single cell values in Figure 1B it is apparent that this difference is not due to an increase in ratio values for all BK+ neurons. Rather the incidence of higher ratio values is greater in the BK+ population, evident as an increase in the interquartile distance (Figure 1B). A quantitative estimate of the incidence of cells for which the PS2/PS1 ratio increased was established by defining a threshold value above which cells were deemed to be sensitized to PS. To establish this threshold, the distribution of all control PS2/PS1 ratio values was plotted for 587 BK– DRG neurons as shown in Figure 1C. The lower end of this distribution of PS2/PS1 ratios was well fit by a Gaussian function (solid line, Figure 1C) and as such the fit parameters were used to define a threshold for sensitization, nominally at the mean plus three standard deviations (0.76 + 3*0.4, broken vertical line, Figure 1C). To determine the incidence of cells with ratio values above this threshold a cumulative frequency distribution (CFD) of PS2/PS1 values was constructed for the control BK– dataset (Figure 1C inset). From this plot, the relative incidence of ratio values exceeding threshold can be readily determined as 7.5% (44/587, vertical broken line, Figure 1C inset). Applying the same procedure to ratio values from BK+ cells (Figure 1D), the number of cells above the sensitization threshold of 2.0 was 39.5% (17/43) and this represents a significantly higher incidence than that for the BK– group [χ^2^_(1,__259)_ = 37.18, *p* < 0.001; Figure 1D]. Similarly, when BK was applied between the second and third PS application (Figure 1E), the comparison of the PS3/PS2 ratio values of the BK+ and BK– groups indicated that the PS3/PS2 ratio for BK+ neurons was significantly higher than for BK– neurons (*p* < 0.01, Mann-Whitney U; Figure 1H). The incidence of cells with PS3/PS2 ratio values above threshold in the BK+ group was 43.4% (23/53) and thus also significantly elevated [χ^2^_(1,__318)_ = 26.99, *p* < 0.01; Figure 1I]. Importantly, for this set of BK+ and BK– neurons, no difference in PS2/PS1 ratio values was apparent in the absence of intervening BK [*p* ≥ 0.05, Mann-Whitney U, Figure 1F; χ^2^_(1,__318)_ = 0.68, *p* = 0.409, Figure 1G]. This indicated that BK sensitized PS responses irrespective of the sequence of application (Figures 1B,D,F–I) and established that TRPM3-mediated calcium response amplitudes can be increased by intervening BK in a subset of DRG neurons.

### Functional Characterization of PS+/BK+ Sensory Neurons

**FIGURE 2:**
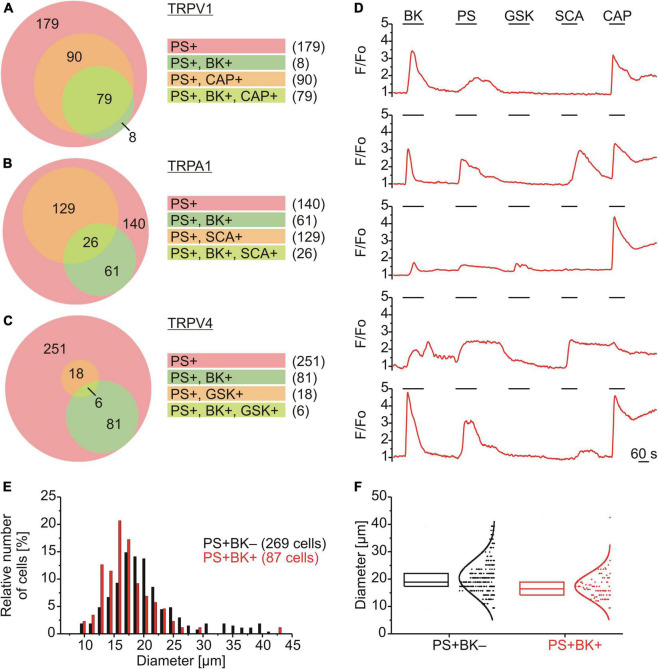
Functional characterization and size distributions of neurons responding to PS. **(A–D)** DRG neurons were stimulated sequentially with bradykinin (BK; 0.5 μM), pregnenolone sulfate (PS; 40 μM), GSK1016790A (TRPV4 agonist; GSK; 1 μM), supercinnamaldehyde (TRPA1 agonist; SCA; 1 μM) and capsaicin (TRPV1 agonist; CAP; 0.5 μM). Values are taken from neurons across eight measurements with cells from one mouse. Euler diagrams represent the relative incidence of cells responding to BK and CAP **(A)**, SCA **(B)**, and GSK **(C)** amongst PS responsive neurons. **(D)** Representative calcium traces from five individual cells. **(E,F)** BK-sensitive and BK-insensitive TRPM3-expressing DRG neurons have similar size distributions. The distributions of sizes (expressed as Feret’s diameter) of the cells analyzed in **(A–C)** are shown in a histogram with a bin size of 1.5 μm **(E)** and as box plot with boxes indicating quartiles and centerline median **(F)**. Each dot-like symbol represents the diameter of one cell and curves represent fits by Gaussian functions.

**FIGURE 3:**
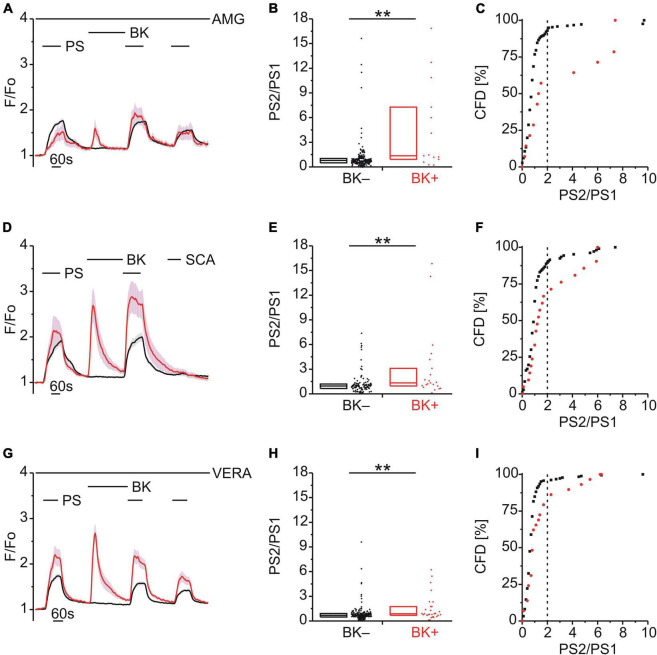
TRPM3 sensitization following BK does not involve TRPV1, TRPA1 or CaV. **(A–C)** Calcium signals from DRG neurons **(A)** during repeat application of pregnenolone sulfate (PS; 40 μM) with bradykinin (BK; 0.5 μM) applied between PS1 and PS2 and in the continuous presence of the TRPV1 inhibitor AMG9810 (AMG; 4 μM). Cells that showed an increase in calcium to BK are indicated in red (BK+; **A–C**; 14 neurons) and those that did not in black (BK–; **A–C**; 216 neurons). PS2/PS1 ratios are shown as a simple distribution (**B**, boxes indicate quartiles and centerline median) and cumulative frequency distribution (CFD; **C**). Each dot-like symbol in **(B,C)** represents the ratio value calculated for a single, individual cell. Values derive from seven measurements with cells from two mice. **(D–F)** Calcium signals from DRG neurons **(D)** during application of PS twice with BK applied between PS1 and PS2 and the TRPA1 agonist SCA (1 μM) applied last of all to identify and exclude TRPA1+ cells. BK+ cells are indicated in red (**D–F**; 21 neurons) and BK– cells in black (**D–F**; 106 neurons). PS2/PS1 ratios are shown as a simple distribution **(E)** and CFD **(F)**. Values derive from four measurements with cells from one mouse. **(G–I)** Calcium signals from DRG neurons **(G)** during repeat application of PS with BK applied between PS1 and PS2 and in the continuous presence of the CaV inhibitor verapamil (VERA; 20 μM). BK+ cells are indicated in red (**G–I**; 29 neurons) and BK– cells in black (**G–I**; 185 neurons). PS2/PS1 ratios are shown as a simple distribution **(H)** and CFD **(I)**. Values derive from six measurements with cells from one mouse. BK+ and BK– groups **(B,E,H)** were compared with Mann-Whitney *U*-tests. **P < 0.01.

The subpopulation of DRG neurons responding to PS was characterized functionally using chemical agonists to assess co-expression of TRPV1 (capsaicin; CAP; 0.5 μM; Figure 2A), TRPA1 (supercinnamaldehyde; SCA; 1 μM; Figure 2B) and TRPV4 (GSK1016790A; GSK; 1 μM; Figure 2C). Of 356 neurons that responded with an increase in calcium to PS, 87 also responded to BK (∼24%) and amongst these PS+/BK+ DRG neurons ∼91% responded to CAP, ∼30% to SCA and ∼7% to GSK. For DRG neurons responding to PS but not to BK, only ∼33% responded to CAP, ∼48% to SCA and ∼7% to GSK (Figure 2). This suggests that the majority of PS+/BK+ DRG neurons also expressed TRPV1 (∼91%), while approximately one third expressed functional TRPA1 and less than 10% TRPV4. The size of TRPM3-expressing DRG neurons showed that BK-sensitive and BK-insensitive cells are small to medium-size neurons with largely overlapping distributions (Figures 3E,F).

### Bradykinin Enhancement of Pregnenolone Sulfate Calcium Response Is Independent of TRPV1, TRPA1, or Voltage-Dependent Calcium Channel Activity

Since the majority of DRG neurons that responded to PS and BK also responded to CAP (Figure 2A), we considered the possibility that the increase in PS responses following BK (Figures 1B,H) may be influenced by the well-established sensitization of TRPV1 by BK (Cesare and McNaughton, 1996; Stucky and Lewin, 1999; Premkumar and Ahern, 2000; Sugiura et al., 2002; Zhang et al., 2008; Hanack et al., 2015; Wang et al., 2015; Mathivanan et al., 2016). To explore this possibility, TRPV1 was blocked with AMG9810 (AMG; 4 μM) but this had no effect on BK-induced TRPM3 sensitization [*p* < 0.01, Mann-Whitney U, Figure 3B; χ^2^_(1,__230)_ = 19.10, *p* < 0.01, Figure 3C], indicating little to no contribution from TRPV1.

BK can also sensitize TRPA1 (Sugiura et al., 2002; Bandell et al., 2004; Bautista et al., 2006; Wang S. et al., 2008; Wang Y. Y. et al., 2008; Chen and Hackos, 2015; Patil et al., 2020) and although only one third of PS+ /BK+ DRG neurons responded to the TRPA1 agonist SCA (Figure 2B), this may also contribute to BK-mediated TRPM3 sensitization. Taking a calcium response to SCA to indicate functional TRPA1 co-expression (Figure 3D), we still observed BK-induced TRPM3 sensitization in neurons not responding to SCA [*p* < 0.01, Mann-Whitney U, Figure 3E; χ^2^_(1,__127)_ = 6.68, *p* < 0.01, Figure 3F], indicating that TRPA1 was not required for BK sensitization.

Finally, the possibility that voltage-dependent calcium channels (CaVs) may contribute to increases in PS calcium signals following BK was examined using the CaV blocker verapamil (VERA; 20 μM; Figure 3G). However, the presence of VERA had no effect on BK-induced TRPM3 sensitization [*p* < 0.01, Mann-Whitney U, Figure 3H; χ^2^_(1,__214)_ = 10.98, *p* < 0.01, Figure 3I], indicating little to no contribution from CaVs.

### Role of Calmodulin-Dependent Protein Kinase II and Protein Kinase C in Bradykinin Enhancement of Pregnenolone Sulfate Calcium Response

**FIGURE 4:**
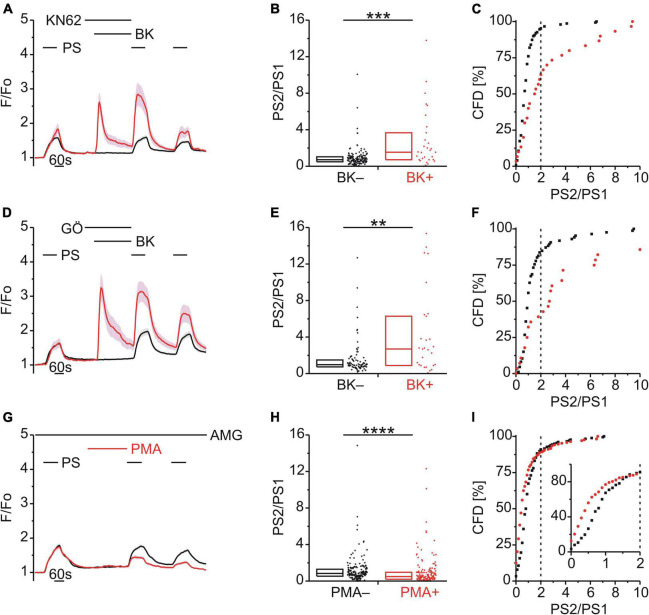
BK-induced TRPM3 sensitization does not involve CaMKII or PKC. **(A–C)** Calcium signals from DRG neurons **(A)** during repeat application of pregnenolone sulfate (PS; 40 μM) with bradykinin (BK; 0.5 μM) applied between PS1 and PS2 in the presence of the CaMKII inhibitor KN62 (1 μM). Cells that showed an increase in calcium to BK are indicated in red (BK+; **A–C**; 30 neurons) and those that did not in black (BK–; **A–C**; 150 neurons). PS2/PS1 ratios are shown as a simple distribution (**B**, boxes indicate quartiles and centerline median) and cumulative frequency distribution (**C**; CFD). Each dot-like symbol in **(B,C)** represents the ratio value calculated for a single, individual cell. Values derive from three measurements with cells from one mouse. **(D–F)** Calcium signals from DRG neurons **(D)** during repeat application of PS with BK applied between PS1 and PS2 in the presence of the PKC inhibitor Gö6983 (GÖ; 1 μM). BK+ cells are indicated in red (**D–F**; 28 neurons) and BK– in black (**D–F**; 86 neurons). PS2/PS1 ratios are shown as a simple distribution **(E)** and CFD **(F)**. Values derive from three measurements with cells from one mouse. **(G–I)** Calcium signals from DRG neurons **(G)** during repeat application of PS either with or without the PKC activator phorbol-12-myristate-13-acetate (PMA; 1 μM) applied between PS1 and PS2 and all the while in the presence of the TRPV1 inhibitor AMG9810 (AMG; 4 μM). Cells that were treated with PMA (PMA+; 168 neurons) are indicated in red and those untreated with PMA (PMA–; 178 neurons) are shown in black. PS2/PS1 ratios are shown as a simple distribution **(H)** and CFD **(I)**. The inset in **(I)** illustrates a zoomed in segment of the CFD for ratio values between 0 and 2. One ratio value (“34.78”) of the PMA+ group was not shown in **(H)** for better visibility, but was included in the analysis. Values derive from eight measurements with cells from two mice. BK+ and BK– as well as PMA+ and PMA– groups **(B,E,H)** were compared with Mann-Whitney *U*-tests. **P < 0.01, ***P < 0.001, ****P < 0.0001.

Both TRPA1 and TRPV1 can be phosphorylated and sensitized by calcium–calmodulin-dependent protein kinase II (CaMKII) and protein kinase C (PKC) (Bonnington and McNaughton, 2003; Zhang et al., 2010; Wang et al., 2015; Patil et al., 2020). Since TRPM3 proteins also harbor several potential phosphorylation sites (Becker et al., 2021), we examined the effect of inhibitors of either CaMKII (KN62; 1 μM; Figure 4A) or PKC (Gö6983; GÖ; 1 μM, Figure 4D) on TRPM3 sensitization by BK. Interestingly, neither inhibition of CaMKII (*p* < 0.001, Mann-Whitney U; Figure 4B) nor PKC (*p* < 0.01, Mann-Whitney U; Figure 4E) prevented TRPM3 sensitization. PS responses were still enhanced by BK as indicated by the elevated incidence of PS2/PS1 ratios with KN62 [χ^2^_(1,__180)_ = 28.44, *p* < 0.01; Figure 4C] and GÖ [χ^2^_(1,__114)_ = 21.07, *p* < 0.01; Figure 4F], respectively. In addition, the PKC activator phorbol-12-myristate-13-acetate (PMA; 1 μM) did not produce an increase in subsequent PS response amplitudes (Figure 4G) or in the incidence of PS2/PS1 ratio values above threshold [χ^2^_(1,__346)_ = 0.129, *p* = 0.719; Figure 4I]. On the contrary, the PS2/PS1 ratios were reduced by PMA (*p* < 0.0001, Mann-Whitney U; Figure 4H), in the presence of AMG9810 to limit PMA effects on TRPV1. This finding indicates a possible reduction in TRPM3 sensitivity to PS (Figures 4G–I). Taken together, neither CaMKII nor PKC inhibition affected sensitization of TRPM3 by BK, while direct, GPCR-independent PKC activation resulted in de-sensitization of TRPM3.

### Diacylglycerol Kinase but Not Diacylglycerol Lipase Contributed to Bradykinin Enhancement of Pregnenolone Sulfate Calcium Responses

**FIGURE 5:**
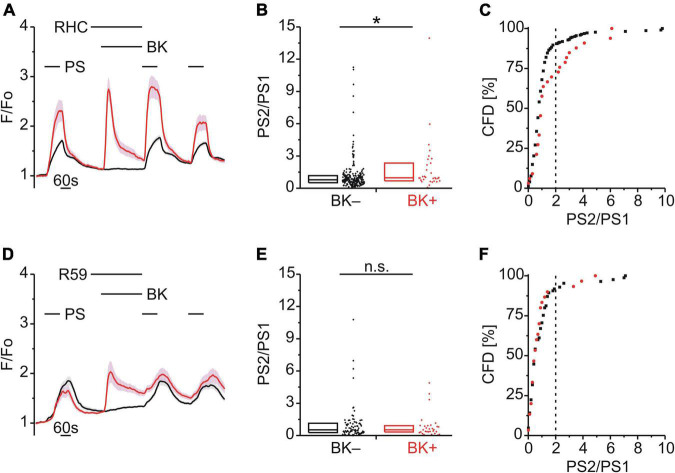
BK-induced TRPM3 sensitization is unaffected by DAG lipase but requires DAG kinase. **(A–C)** Calcium signals from DRG neurons **(A)** during repeat application of pregnenolone sulfate (PS; 40 μM) with bradykinin (BK; 0.5 μM) applied between PS1 and PS2 and in the presence of the diacylglycerol (DAG) lipase inhibitor RHC-80267 (RHC; 10 μM). Cells that showed an increase in calcium to BK are indicated in red (BK+ ; **A–C**; 33 neurons) and those that did not in black (BK–; **A–C**; 222 neurons). PS2/PS1 ratios are shown as a simple distribution (**B**, boxes indicate quartiles and centerline median) and cumulative frequency distribution (**C**; CFD). Each dot-like symbol in **(B,C)** represents the ratio value calculated for a single, individual cell. One BK+ ratio value (“17.9”) in **(B)** is not displayed for better visibility but was included in the analysis. Values derive from four measurements with cells from one mouse. **(D–F)** Calcium signals from DRG neurons **(D)** during repeat application of PS with BK applied between PS1 and PS2 and in the presence of the DAG kinase inhibitor R-59-022 (R59; 10 μM). BK+ cells are indicated in red (**D–F**; 30 neurons) and BK– cells in black (**D–F**; 85 neurons). PS2/PS1 ratios are shown as a simple distribution **(E)** and CFD **(F)**. Values derive from six measurements with cells from two mice. BK+ and BK– groups **(B,E)** were compared with Mann-Whitney *U*-tests. *P < 0.05; n.s., not significant.

In DRG neurons, Gq-coupled BKR activation leads to PLC activity and increased levels of diacylglycerol (DAG) and inositol 1,4,5-trisphosphate (IP_3_). To explore a possible role for DAG in TRPM3 sensitization, BK was applied together with either the DAG lipase (DAGL) inhibitor RHC-80267 (RHC; 10 μM; Figure 5A) or the DAG kinase (DAGK) inhibitor R-59-022 (R59; 10 μM; Figure 5D). While inhibition of DAGL did not affect BK-induced TRPM3 sensitization [*p* < 0.05, Mann-Whitney U, Figure 5B; χ^2^_(1,__255)_ = 10.14, *p* < 0.01, Figure 5C], inhibition of DAGK did [*p* ≥ 0.05, Mann-Whitney U, Figure 5E; χ^2^_(1,__115)_ = 0.087, *p* = 0.768, Figure 5F].

### Calcium Store Release Enhanced Pregnenolone Sulfate Calcium Responses

**FIGURE 6:**
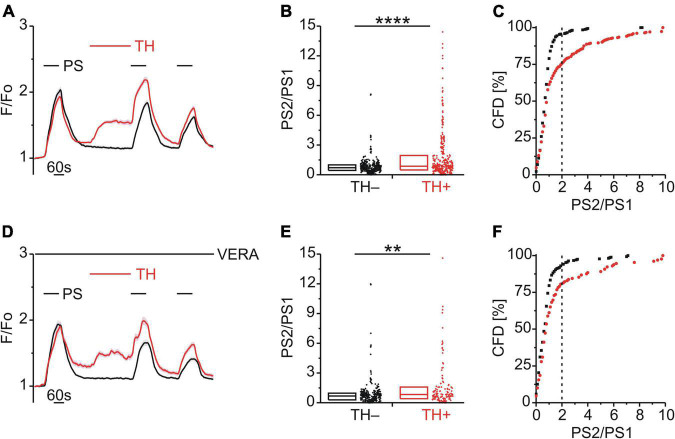
Store released calcium sensitizes TRPM3. **(A–C)** Calcium signals from DRG neurons **(A)** during repeat application of pregnenolone sulfate (PS; 40 μM) and with or without the SERCA inhibitor thapsigargin (TH; 2 μM) applied between PS1 and PS2. Cells that were treated with TH are indicated in red (TH+; **A–C**, 336 neurons) and those untreated with TH are shown in black (TH–; **A–C**, 319 neurons). PS2/PS1 ratios are shown as a simple distribution (**B**, boxes indicate quartiles and centerline median) and cumulative frequency distribution (**C**; CFD). Two TH+ ratio values (“16.4” and “19.1”) in **(B)** are not displayed for better visibility but were included in the analysis. Each dot-like symbol in **(B,C)** represents the ratio value calculated for a single, individual cell. Values derive from 12 measurements with cells from four mice. **(D–F)** Calcium signals from DRG neurons **(D)** during repeat application of PS and with or without TH applied between PS1 and PS2 and all the while in the presence of the CaV inhibitor verapamil (VERA; 20 μM). Cells that were treated with TH are indicated in red (TH+; **D–F**, 129 neurons) and those untreated with TH are shown in black (TH–; **D–F**, 270 neurons). Two TH+ ratio values (“18.2” and “26.3”) in **(E)** are not displayed for better visibility but were included in the analysis. Values derive from eight measurements with cells from two mice. TH+ and TH– groups **(B,E)** were compared with Mann-Whitney *U*-tests. **P < 0.01, ****P < 0.0001.

Having identified a possible role of DAG phosphorylation in BK-induced TRPM3 sensitization, the role of IP_3_ was examined. Interestingly, an early study utilizing HEK-293 cells expressing recombinant TRPM3 had shown that blockade of the sarco/endoplasmic reticulum calcium ATPase (SERCA) with thapsigargin (TH) augmented TRPM3-mediated calcium entry (Lee et al., 2003). Consistent with this finding, TH (2 μM; Figure 6A) increased the PS calcium responses (*p* < 0.0001, Mann-Whitney U; Figure 6B) and the incidence of cells with an elevated PS2/PS1 ratio [χ^2^_(1,__655)_ = 52.41, *p* < 0.01; Figure 6C]. These effects persisted in the presence of VERA to block CaVs [*p* < 0.01, Mann-Whitney U, Figure 6E; χ^2^_(1,__399)_ = 18.55, *p* < 0.01, Figure 6F], indicating a lack of involvement of CaV channels.

### Exocytosis Contributed to Bradykinin Enhancement of Pregnenolone Sulfate Calcium Responses

**FIGURE 7:**
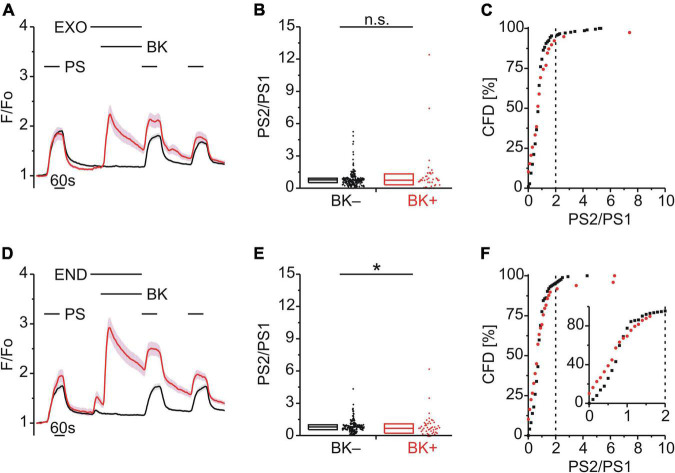
BK-induced TRPM3 sensitization is strongly affected by exocytosis inhibitors. **(A–C)** Calcium signals from DRG neurons **(A)** during repeat application of pregnenolone sulfate (PS; 40 μM) with bradykinin (BK; 0.5 μM) applied between PS1 and PS2 and in the presence of the exocytosis inhibitor exo1 (EXO; 20 μM). Cells that showed an increase in calcium to BK are indicated in red (BK+; **A–C**; 39 neurons) and those that did not in black (BK–; **A–C**; 213 neurons). PS2/PS1 ratios are shown as a simple distribution (**B**, boxes indicate quartiles and centerline median) and cumulative frequency distribution (**C**; CFD). Each dot-like symbol in **(B,C)** represents the ratio value calculated for a single, individual cell. Values derive from six measurements with cells from two mice. **(D–F)** Calcium signals from DRG neurons **(D)** during repeat application of PS with BK applied between PS1 and PS2 and in the presence of the exocytosis inhibitor endosidin 2 (END; 10 μM). BK+ cells are indicated in red (**D–F**; 49 neurons) and BK– cells in black (**D–F**; 174 neurons). PS2/PS1 ratios are shown as a simple distribution **(E)** and CFD **(F)**. The inset in **(I)** illustrates a zoomed in segment of the CFD for ratio values between 0 and 2. Values derive from six measurements with cells from two mice. BK+ and BK– groups **(B,E)** were compared with Mann-Whitney *U*-tests. *P < 0.05; n.s., not significant.

Enhanced PS responses could arise through a calcium-dependent increase in TRPM3 surface expression mediated by vesicular exocytosis. To explore a possible role of exocytosis in TRPM3 sensitization, BK was applied together with either exo1 (EXO; 20 μM; Figure 7A) or endosidin 2 (END; 10 μM; Figure 7D), both inhibitors of exocytosis. The PS2/PS1 ratio did not increase following EXO [*p* ≥ 0.05, Mann-Whitney U, Figure 7B; χ^2^_(1,__252)_ = 0.605, *p* = 0.437, Figure 7C] and similarly the PS2/PS1 ratio did not increase following END but rather was reduced significantly for BK+ cells [*p* < 0.05, Mann-Whitney U, Figure 7E; χ^2^_(1,__223)_ = 0.068, *p* = 0.795, Figure 7F].

The ability of elevated intracellular calcium to emulate the sensitizing effect of BK on PS responses prompted us to examine potential correlations between preceding calcium levels in response to BK or TH and PS2/PS1 ratios. For cells showing a calcium response to BK, linear regression of the PS2/PS1 ratio on the BK response indicated no correlation [*r*(43) = 0.08, *r*^2^ = 0.006]. Similarly, there was no correlation between the PS2/PS1 ratio and the amplitude of the calcium response to TH [*r*(314) = 0.05, *r*^2^ = 0.0024]. A lack of correlation in both cases suggests that the level of cytosolic calcium is not predictive of the ensuing change in PS response amplitude following either BK or TH. Comparisons of the BK-induced calcium response amplitudes revealed that the DAG kinase inhibitor R59 (Figures 5D–F) as well as the TRPV1 inhibitor AMG9810 (Figures 2A–C) significantly reduced the BK-induced calcium response amplitudes when tested against amplitudes obtained under control conditions (Figure 3). However, as described above, we did not find a correlation between the BK response amplitude and the post-BK PS response amplitude and the fraction of cells within the PS+ population that were also BK+ was not smaller in the R59 experiment in comparison to the RHC experiment (Figure 5A) nor other experiments. Moreover, Figure 2A illustrates that in the presence of the TRPV1 inhibitor AMG9810, the BK-induced calcium response amplitudes were also smaller, yet an increase in the second PS response was still apparent (Figures 2A–C). This further corroborates that the level of cytosolic calcium during BK is not predictive of the ensuing changes in PS response amplitudes. We have not further investigated the mechanism(s) contributing the R59 and AMG9810 mediated reductions in BK responses. Supplementary Figure 1 illustrates the amplitude of BK-induced calcium responses in the presence of all the substances used throughout the study.

Overall, we show here that a preceding calcium response to BK leads to an increase in the amplitude of TRPM3-mediated calcium responses to PS in a subset of adult mouse DRG neurons. TRPM3 sensitization induced by BK was attributed to lingering effects of the preceding increase in intracellular calcium and could be prevented by inhibition of DAGK and exocytosis.

## Discussion

TRPM3 is involved in the development of inflammatory hyperalgesia to heat (Vriens et al., 2011; Mulier et al., 2020) and we demonstrate here that TRPM3 is sensitized by BK in a subpopulation of DRG neurons isolated acutely from adult mice. Within the subpopulation of DRG neurons that responded to BK with an increase in intracellular calcium, PS calcium responses were augmented after BK and most of these PS+/BK+ neurons also responded to the TRPV1 agonist CAP. Inhibition of DAGK and exocytosis prevented BK-induced sensitization of PS responses, while PS sensitization was also observed following increases in intracellular calcium via TH-mediated store release. However, inhibition of PKC did not prevent BK-induced sensitization while phorbol ester activation of PKC even reduced PS responses. This indicates that DAGK and calcium exert positive regulatory roles in TRPM3 sensitization, while elevated DAG and PKC activity have negative regulatory effects on TRPM3. Targeting TRPM3 sensitization may thus be a potential rationale for the development of analgesic strategies in the context of inflammatory hypersensitivity.

Calcium responses to PS, BK, and CAP indicated co-expression of functional TRPV1 in ∼91% of TRPM3+/BKR+ cells (Figure 3A). Since TRPM3, TRPV1, and BKR expression does not decline appreciably during the first days in culture (Wangzhou et al., 2020), this functionally defined TRPM3+/BKR+/TRPV1+ subpopulation is consistent with subgroup S3 of small diameter DRG neurons identified by transcriptional profiling (Patil et al., 2020). The S3 neuronal population has elevated transcript levels for CGRP, BKR2, and TRPA1, but not MRGPRA3, MRGPRD, or vGLUT3. The co-expression of CGRP with TRPM3 in this neuronal group is consistent with reports of CGRP release from mouse hindpaw skin in response to PS activation of TRPM3 (Held et al., 2015).

### Signaling Pathways Involved in Bradykinin-Induced Transient Receptor Potential Channel Melastatin 3 Sensitization

BK plays an important role in the development of inflammatory heat hyperalgesia (Manning et al., 1991; Dray and Perkins, 1993), acting via Gq/PLC, Gi/phospholipase A2 and Gs/adenylate cyclase pathways to sensitize for example TRPV1 or TRPA1 (Petho and Reeh, 2012). TRPV1 can be sensitized by diverse inflammatory mediators like BK, ATP, NGF, and prostaglandins coupling to different pathways (Huang et al., 2006). Excitation of cardiac sensory neurons by BK, for example, involved 12-lipoxygenase products, IP_3_, elevated intracellular calcium and activation of TRPV1 (Wu and Pan, 2007). TRPA1 was activated by BK in CHO cells and cultured DRG neurons via a pathway involving PLC (Bandell et al., 2004). BK could also sensitize TRPA1 in HEK-293 cells as well as in sensory neurons and this sensitization could be mimicked by PLC or protein kinase A (PKA) activation and blocked by PLC or PKA inhibition (Wang S. et al., 2008). However, BK-evoked nociceptor responses cannot be fully explained by TRPV1 or TRPA1 activation or sensitization, since for example BK-induced C-fiber excitation in wildtype mice was comparable to that in TRPV1-KO mice. In addition, nocifensive behavior of wildtype mice after injection of a high dose (1 nmol/site) of BK was indistinguishable from that of TRPV1-KOs (Katanosaka et al., 2008).

**FIGURE 8:**
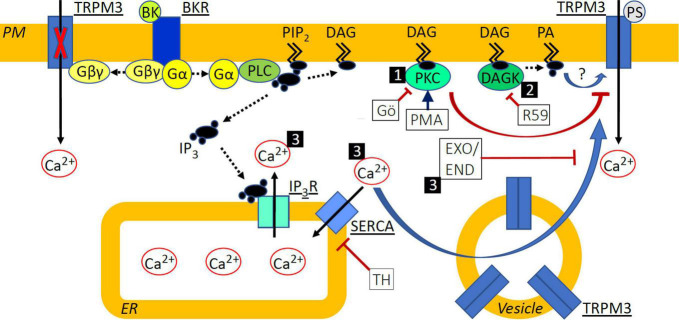
Schematic indicating signal transduction pathways deriving from BKR activation and affecting TRPM3 sensitization. (1) Protein Kinase C (PKC) activation with phorbol-12-myristate-13-acetate (PMA) de-sensitized TRPM3 responses to pregnenolone sulfate (PS), while inhibition with Gö6983 (GÖ) did not. (2) Diacylglycerol (DAG) kinase (DAGK) inhibition with R-59-022 (R59) prevented sensitization of TRPM3 by bradykinin (BK) and we speculate that this effect could be mediated by a reduction in the DAG metabolite phosphatidic acid (PA) or by accumulated DAG, directly influencing TRPM3 function in a positive or negative fashion, respectively. (3) Elevated cytosolic calcium (Ca^2+^) via inhibition of SERCA with thapsigargin (TH) or following BK receptor (BKR) activation sensitized TRPM3 to PS and increased exocytosis of vesicular TRPM3 may contribute to this. Inhibition of exocytosis with either exo1 (EXO) or endosidin 2 (END) prevented sensitization of TRPM3 by BK and we hypothesize that this effect could be explained by the prevention of TRPM3 accumulation at the cell surface. PM, plasma membrane; PIP_2_, phosphatidyl inositol bisphosphate; Gα/βγ, G protein α/βγ subunit; PLC, phospholipase C; IP_3_, inositol trisphosphate; IP_3_R, IP_3_ receptor; ER, endoplasmic reticulum. Solid black arrows indicate Ca^2+^ movement; dashed black arrows indicate translocation or modification of molecules; blue arrows indicate activation or sensitization; red arrows indicate inhibition or de-sensitization.

BK also modulates the activity of CaV channel isoforms. N-type CaVs, for example, were inhibited by BK via sequential activation of G13 and Rac1/Cdc42 G proteins as well as p38 mitogen-activated protein kinase in NG108-15 cells (Wilk-Blaszczak et al., 1998). Moreover, BK inhibited N-type CaVs in sympathetic neurons by inducing PIP_2_ hydrolysis, but only when PIP_2_ re-synthesis, neuronal calcium sensor-1 or IP_3_ receptors were blocked (Gamper et al., 2004). The BK-induced inhibition of calcium currents in superior cervical ganglion neurons was also attributed to the hydrolysis of PIP_2_ by PLC but not to PLC downstream targets (Lechner et al., 2005). BK-induced inhibition of L-type CaVs in NG108-15 cells, however, resulted from reduced probability of channel opening, was mimicked by the DAG analog 1,2-oleoyl-acetyl glycerol and blocked by PKC inhibitors (Toselli and Taglietti, 2005). Overnight treatment of cultured DRG cells with BK did not affect the peak T-type current amplitude but increased the subpopulation of neurons expressing functional T-type CaVs and this effect was abolished by PLC inhibition (Huang et al., 2016). Here we show that when applied without overlap, BK can also sensitize TRPM3 to PS (Figure 1) via DAGK (Figures 5D–F) with a possible contribution from calcium store release (Figure 6) and exocytosis (Figure 7). Agonist binding to both BKR1 and BKR2 leads to DAG and IP_3_ liberation from PIP_2_ via Gq-mediated PLC activation, and thus release of calcium from intracellular stores and activation of several kinases including PKC (Couture et al., 2001; Huang et al., 2006; Petho and Reeh, 2012). Although the PLCβ-PKCε-TRPV1 axis was found to underlie BK-induced sensitization of TRPV1 to heat (Cesare and McNaughton, 1996; Cesare et al., 1999; Sugiura et al., 2002) neither blockade of PKC nor blockade of CaMKII affected BK-induced sensitization of PS responses (Figures 4A–F). Moreover, direct, GPCR-independent PKC activation by PMA resulted in reduced PS calcium response amplitudes (Figures 4G–I, 8, pathway 1). Interestingly, BK has been shown to downregulate TRPM8 and upregulate TRPV1 via PKC and the PKC-dependent effect on TRPM8 comprised both a reduced channel conductance and a reduction in the number of channels at the cell surface (Premkumar et al., 2005). It is tempting to speculate that increased PKC activity may regulate TRPV1 positively, while exerting a negative regulatory influence on TRPM3 in DRG neurons similar to that reported for TRPM8.

Inhibition of DAGK prevented BK-induced enhancement of PS calcium responses (Figure 5). DAGK catalyzes the reaction from DAG to phosphatidic acid while DAGL catalyzes the hydrolysis of DAG to monoacylglyerol and a free fatty acid. Interestingly, DAG levels have been shown to be differentially regulated by DAGK and DAGL in an overexpression system, where only inhibition of DAGK increased DAG levels, indicating that DAGK but not DAGL activity limits DAG accumulation (Liu et al., 2020). Accordingly, it is possible that inhibition of DAGK results in DAG accumulation in sensory neurons and this leads to increased PKC activity and subsequent abrogation of BK-induced sensitization of TRPM3. It is therefore possible that phosphatidic acid is involved in BK-induced sensitization of PS responses (Figure 8, pathway 2).

TRPM3 can be regulated by changes in intracellular calcium. Elevated intracellular calcium levels, via prolonged TRPM3 stimulation or by flash-induced photolysis of caged calcium, either de-sensitized or inhibited channel activity (Vriens et al., 2011, 2014; Przibilla et al., 2018). In contrast, TRPM3-mediated calcium signals in transfected HEK-293 cells were enhanced following Gq-coupled muscarinic receptor activation with carbachol or calcium store release by TH (Lee et al., 2003). Interestingly, in our experiments with DRG neurons and native TRPM3 channels, receptor-independent calcium store release was sufficient to sensitize PS responses (Figure 6). A possibility to account for enhanced PS responses in the presence of elevated calcium would be a calcium-dependent increase in TRPM3 surface expression mediated by exocytosis of intracellular vesicles (Figure 8, pathway 3) known to contain TRPM3 (Ghosh et al., 2016). This process of exocytotic mobilization of channels and receptors has been shown to be relevant, e.g., for BK-induced sensitization of TRPV1 in peptidergic nociceptors, in which large dense core vesicles comprising TRPV1 are trafficked to the neuronal membrane in an αCGRP-dependent manner (Mathivanan et al., 2016). Similarly, in transfected HEK-293 cells, TRPM8 and TRPM3 localize to vesicles for trafficking and incorporation into the plasma membrane (Ghosh et al., 2016). TRPM3 has also been shown to interact with activating transcription factor 4 (ATF4) in DRG neurons. ATF4 mediates membrane accumulation of TRPM3 in response to heat (Xie et al., 2021) but a role for calcium in ATF4-dependent TRPM3 membrane trafficking has not been elucidated. Using exocytosis inhibitors (Figures 7, 8, pathway 3) we show that trafficking of vesicular TRPM3 to the cell surface may contribute to BK-induced TRPM3 sensitization in DRG neurons. Figure 8 summarizes the intracellular signaling pathways that may be involved in TRPM3 regulation.

### Regulation of Transient Receptor Potential Channel Melastatin 3 by G Protein-Coupled Receptors

Gq-coupled GPCRs, including BKRs, activate PLC, which hydrolyzes PIP_2_ into DAG and IP_3_, and TRPM3 requires PIP_2_ for activation and is inhibited by PIP_2_ depletion in planar lipid bilayer as well as in cellular overexpression systems (Badheka et al., 2015; Toth et al., 2015; Uchida et al., 2016). However, the role of PIP_2_ for TRPM3 in neurons has not been established. In addition, discriminating between the effects of G proteins and phosphoinositides on ion channel function has been found challenging. For example, PIP_2_-independent (Zhang, 2019) as well as PIP_2_-dependent (Liu et al., 2019) mechanisms have been proposed for GPCR-induced TRPM8 inhibition. In native DRG neurons, TRPM3 is co-expressed with a variety of GPCRs and their co-activation can lead to channel inhibition via Gβγ (Badheka et al., 2017; Dembla et al., 2017; Quallo et al., 2017; Masuho et al., 2018; Touhara and MacKinnon, 2018; Alkhatib et al., 2019). Gβγ binds directly to a sequence of 10 amino acids in exon 17 of TRPM3, and since exon 17 is subject to alternative splicing, this region constitutes an on–off switch for TRPM3 inhibition (Oberwinkler and Philipp, 2014; Behrendt, 2019; Behrendt et al., 2020). Our finding that BKR activation sensitized subsequent PS responses (Figure 1) appears to contrast with a recent report by Alkhatib et al. (2019) showing inhibition of PS-induced calcium responses during concomitant BK application in DRG neurons (Alkhatib et al., 2019). Importantly, in the Alkhatib study, calcium response inhibition by BK resulted from liberation of Gβγ and consistent with this, both the onset and offset kinetics of PS response suppression were rapid (Alkhatib et al., 2019). In the experiments reported here, BK was applied in the interval preceding PS, such that at the time of the subsequent PS application, little if any Gβγ liberated during the BK stimulation would be available for TRPM3 inhibition. In this study and that of Alkhatib et al. (2019) cultured mouse DRG neurons were used. We defined PS+/BK+ DRG neurons functionally by their calcium responses to PS and BK applied separately (∼17%, Figure 1; ∼24%, Figure 3). Alkhatib et al. (2019) found that ∼29% (86 of 293) of DRG neurons were PS+/BK+ as indicated by BK’s ability to suppress a PS response. Accordingly, it is likely that the two PS+/BK+ sensory neuron populations overlap to a large degree. However, while it is known that exon 17 is required for TRPM3 inhibition by GPCR activation (see above), it is not known if this region is also required for BK-induced sensitization or if this effect is sensitive to alternative splicing of TRPM3.

In general, alternative splicing of sensory transduction proteins, ion channels and growth factors has long been recognized as a relevant factor in the development of chronic pain (Donaldson and Beazley-Long, 2016). One example is TRPA1, for which a change in splice variant expression levels in inflammatory as well as neuropathic pain models was observed (Zhou et al., 2013). Moreover, components of the machinery underlying TRPA1 alternative splicing were identified and could be manipulated to promote particular splice events, indicating that targeting alternative splicing could be part of future analgesic strategies (Huang et al., 2021). Splice variants of TRPV1 can be categorized into loss of function, gain of function and mixed based upon their properties as homomeric channels (Wang et al., 2016). Furthermore, TRPV1 variants can form heteromeric channels and the exact composition determines expression and function of the complex (Schumacher and Eilers, 2010). For TRPM3, splice variant expression in mouse tissue has only been determined systematically in the choroid plexus revealing 17 variants and six splice sites, including exon 17 (Frühwald et al., 2012). In addition, four distinct, exon 17-containing, TRPM3 variants have been identified in mouse DRGs and subsequently characterized functionally in overexpression systems, revealing differences in responses to chemical and physical stimuli (Uchida et al., 2019). These variants differ in exon 15 and most of exon 28. The presence or absence of the amino acids encoded by these exons might play a role in BK-induced TRPM3 sensitization, for example through an ability to interact with calcium or phosphatidic acid. Such a “sensitization motif” may then be dominant over the exon 17-“inhibition motif.”

TRPM3 is implicated in the development of heat hyperalgesia that commonly accompanies inflammatory pain states, such as arthritis. In this role, TRPM3 can be affected by intracellular enzymes and direct binding of G protein subunits set in motion by binding of inflammatory mediators to their cognate receptors. Although inflammation leads to an increased sensitivity to heat, intracellular regulation of TRPM3 leads to an inhibition of the channel. Here we demonstrate that TRPM3 can also be sensitized by inflammatory mediators, specifically BK, acting on the same cell. TRPM3 sensitization was only observed if BK application preceded TRPM3 stimulation and DAGK, elevated cytosolic calcium as well as vesicular exocytosis each contributed to this TRPM3 sensitization. An increase in the sensitivity of TRPM3 following exposure to BK may represent a pathway whereby elevated surface expression of TRPM3 contributes to altered heat sensitivity during inflammation.

## Materials and Methods

### Animals

For isolation and culture of DRG neurons we used adult male C57BL/6 mice, which were anesthetized with volatile anesthetic before being killed by cervical dislocation. Mice were housed in ventilated polycarbonate cages in a controlled environment with access to food and water *ad libitum*. Approval for animal use was provided under I-19/05 at the Medical Faculty Mannheim, Heidelberg University.

### Isolation and Culture of Dorsal Root Ganglion Neurons

Techniques for isolation and DRG cell culturing were similar to those previously described (Dembla et al., 2017). Briefly, DRGs were harvested from all segments and placed into chilled HBSS (Thermo Fisher Scientific, Karlsruhe, Germany). Isolated ganglia were partially digested for 40 min in 1.8 U/ml liberase DH Research Grade (Roche, Mannheim, Germany) at 37°C and digestion was stopped by adding 1 ml culture medium consisting of DMEM, 10% FBS and 1x penicillin-streptomycin (all from Thermo Fisher Scientific, Karlsruhe, Germany). DRGs were triturated with a 1,000 μl pipette and washed once with culture medium. After centrifugation, the supernatant was discarded, and the cells were suspended in culture medium. Subsequently, one tenth to one twelfth of the cell suspension was plated onto the center of a 35 mm plastic dish pre-coated with laminin (Sigma-Aldrich, Munich, Germany). The cells were left to adhere at 37°C in an incubator in a humidified atmosphere containing 5% CO_2_ for 1 h after which 2 ml of culture medium were added. Cells were used for measurements on the following day.

### Fluorescence Calcium Imaging

Isolated DRG neurons were incubated with 3 μM Fluo8-AM (1 mM stock in DMSO, AAT Bioquest, Sunnyvale, CA, United States) for 40 min in culture medium at room temperature (RT) in the dark. After loading, cells were placed on the microscope stage and perfused continuously with extracellular solution at RT. A gravity-driven system was used for perfusion. Images were acquired using a back-illuminated 512 * 512 pixel cooled EMCCD camera (Evolve 512, Photometrics, Tucson, AZ, United States). The camera was connected to the side port of an inverted microscope (Axiovert 200, Zeiss, Jena, Germany). Fluorescence images were acquired using a 465 nm LED (Prior Scientific, MA, United States) together with a filter set (excitation BP 450–490 nm, dichroic = 510 nm, emission = 515 nm LP, Chroma Technologies) and μManager software with image acquisition controlled via an Arduino Duemilanove (Watterott electronic, Leinefelde, Germany). Fluorescence image sequences were acquired at either 1 or 0.5 Hz. Images were acquired initially for not less than 2 min to establish baseline conditions before chemicals were superfused onto the cells. At the end of each protocol, solution containing 75 mM potassium was used to identify functional neurons.

### Solutions and Chemicals

Extracellular solution contained (in mM): 145 NaCl, 10 CsCl, 3 KCl, 2 CaCl2, 2 MgCl2, 10 HEPES, 10 D-glucose. Extracellular solution with an elevated concentration of potassium was obtained by replacing 75 mM NaCl with an equimolar amount of KCl. Solutions were adjusted to pH 7.2 with NaOH. Pregnenolone sulfate sodium salt (PS; TRPM3 agonist) and phorbol-12-myristate-13-acetate (PMA; PKC activator) were from Santa Cruz (Heidelberg, Germany). The following substances were purchased from Biomol (Hamburg, Germany): supercinnamaldehyde (SCA; TRPA1 agonist), Gö6983 (GÖ; PKC inhibitor), KN-62 (KN62; CaMKII inhibitor), R-59-022 (R59; DAG kinase inhibitor), RHC-80267 (RHC; DAG lipase inhibitor), exo1 (EXO), and endosidin 2 (END; both inhibitors of exocytosis). The following substances were obtained from Sigma-Aldrich: bradykinin acetate salt (BK; BKR agonist), capsaicin (CAP; TRPV1 agonist), GSK1016790A (GSK; TRPV4 agonist), verapamil hydrochloride (VERA; CaV blocker). Thapsigargin (TH; SERCA inhibitor) was from Sigma and Santa Cruz. AMG9810 (AMG; TRPV1 inhibitor) was obtained from Santa Cruz and Biomol. All chemical reagents were prepared as stock solutions in DMSO, with the exception of BK that was dissolved in acetic acid. The desired concentration of each substance was achieved by diluting stock solution into extracellular perfusing solution on the day of each experiment.

### Data Analysis and Statistics

To monitor changes in calcium fluorescence over time, regions of interest (ROIs) were delineated manually by placing circular fields within cell boundaries of visually identified neurons in ImageJ. Additional ROIs placed in areas outside of cells were used to determine background intensity. Average fluorescence intensity (aF) across all pixels within each ROI was determined and aF values for each ROI were boxcar smoothed (running average of five). Average background fluorescence was subtracted from the time series for each cell ROI to obtain background-subtracted fluorescence values (F). Calcium responses to chemical stimuli were evaluated for each ROI by determining the mean and standard deviation (SD) of F values over the 10 images preceding stimulation (pre-F). A ROI was considered to have responded to a chemical stimulus if the mean F during the stimulation period was greater than the mean + 5*SD during the pre-F period. Values of F were normalized to the first value (F/Fo) and all F/Fo plots indicate means ± SEMs. For protocols using repeat application of PS, the effect of intervening stimuli (i.e., BK, PMA, or TH applied between successive PS exposures) was analyzed as follows: (i) the highest *F*-value during the PS stimulus was determined (resulting in Fmax), (ii) the last *F*-value before the start of the PS application was subtracted from Fmax (resulting in ΔFmax), (iii) the ratio of ΔFmax during the succeeding relative to the preceding PS stimulus was determined (resulting in PS2ΔFmax/PS1ΔFmax and/or PS3ΔFmax/PS2ΔFmax). Then the ratios of the BK-responders (BK+)/PMA-treated/TH-treated cells and the BK-non-responders (BK–)/PMA-non-treated/TH-non-treated cells were compared.

Box plots of PS2ΔFmax/PS1ΔFmax (or PS3ΔFmax/PS2Δ Fmax) ratio values indicate medians (horizontal lines) and 25/75% percentiles (boxes). Individual cells are considered independent biological replicates. In all experiments, the number of biological replicates (determined as described above and referred to as “n”) was used for statistical testing and to calculate SEM values. We additionally report the number of recordings for calcium imaging experiments, which indicates the number of dishes. Outliers were never removed from any data set. Group comparisons were made using non-parametric Mann Whitney *U*-tests in Graphpad Prism (La Jolla, CA, United States), Chi-squared tests were used to assess differences in the incidence of cells with ratio values above threshold. In all cases, we accepted *p*-values smaller than 0.05 as statistically significant. Curve fitting was performed using the Levenberg-Marquardt algorithm for least-squares fitting implemented in Igor Pro (Wavemetrics, Lake Oswego, United States).

## Data Availability Statement

The original datasets are available from the corresponding author upon reasonable request.

## Ethics Statement

The use of animal tissue was reviewed and approved by the Medical Faculty Mannheim, Heidelberg University, Mannheim, Germany, under I-19/05.

## Author Contributions

MB conceptualized and designed the study, prepared and conducted imaging experiments, analyzed the data, wrote, reviewed, and edited the manuscript. HJS and RC analyzed the data, wrote, reviewed, and edited the manuscript. MS wrote, reviewed, and edited the manuscript. All authors contributed with interpretation of the work and to manuscript revision, all read and approved the submitted version.

## Conflict of Interest

The authors declare that the research was conducted in the absence of any commercial or financial relationships that could be construed as a potential conflict of interest.

## Publisher’s Note

All claims expressed in this article are solely those of the authors and do not necessarily represent those of their affiliated organizations, or those of the publisher, the editors and the reviewers. Any product that may be evaluated in this article, or claim that may be made by its manufacturer, is not guaranteed or endorsed by the publisher.

## Supplementary Material

Supplementary Figure 1 BK-induced calcium response amplitudes. The amplitude of BK-induced calcium responses in the presence of all the substances used throughout the study. Scatter dot plot (black and gray) with mean and standard error (red).
