# Supplementary figures and images for: Bradykinin-Induced Sensitization of Transient Receptor Potential Channel Melastatin 3 Calcium Responses in Mouse Nociceptive Neurons
Source: Front Cell Neurosci. 2022 Apr 13;16:843225. doi: 10.3389/fncel.2022.843225 (PMC9043526; doi:10.3389/fncel.2022.843225)

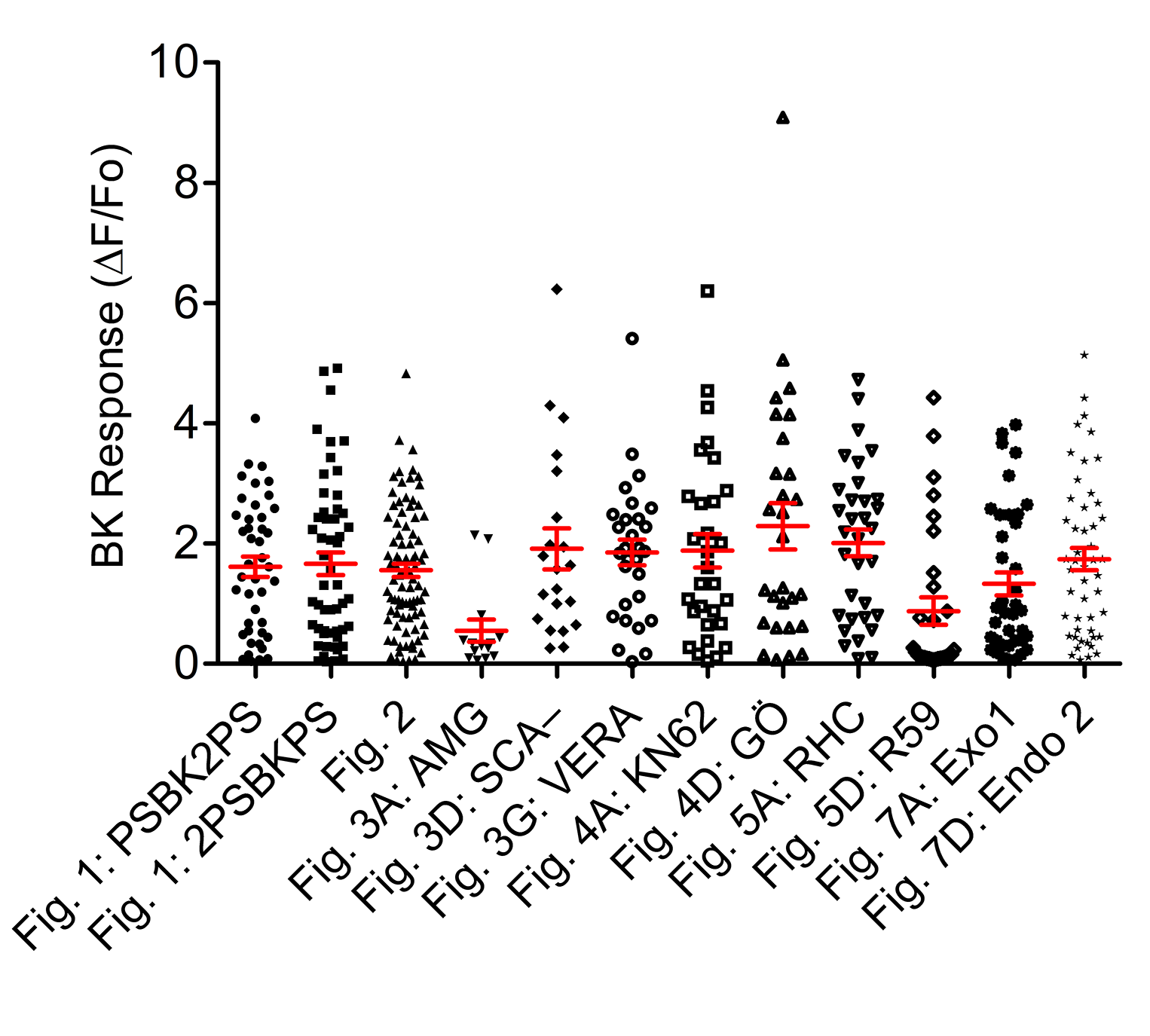

Supplement: Supplementary Figure 1 — BK-induced calcium response amplitudes. The amplitude of BK-induced calcium responses in the presence of all the substances used throughout the study. Scatter dot plot (black and gray) with mean and standard error (red). [file Image_1.tif]
